# Impact of Novel Treatments in Patients with Melanoma Brain Metastasis: Real-World Data

**DOI:** 10.3390/cancers15051461

**Published:** 2023-02-25

**Authors:** Sophie H. A. E. Derks, Joost L. M. Jongen, Edgar L. van der Meer, Li Shen Ho, Cleo Slagter, Arjen Joosse, Maja J. A. de Jonge, Joost W. Schouten, Esther Oomen-de Hoop, Martin J. van den Bent, Astrid A. M. van der Veldt

**Affiliations:** 1 Department of Neuro-Oncology, Erasmus MC Cancer Institute, 3015 GD Rotterdam, The Netherlands; 2 Department of Medical Oncology, Erasmus MC Cancer Institute, 3015 GD Rotterdam, The Netherlands; 3 Department of Radiology & Nuclear Medicine, Erasmus Medical Center, 3015 GD Rotterdam, The Netherlands; 4 Department of Radiotherapy, Erasmus MC Cancer Institute, 3015 GD Rotterdam, The Netherlands; 5 Department of Neurosurgery, Erasmus Medical Center, 3015 GD Rotterdam, The Netherlands

**Keywords:** melanoma, brain neoplasms/metastasis, immunotherapy, immune checkpoint inhibitors, molecular targeted therapy, BRAF/MEK inhibitors, radiotherapy, survival

## Abstract

**Simple Summary:**

Cutaneous melanoma is highly prone to metastasize to the brain, with a historically poor overall survival of only 4–5 months. Over the past decade, novel drugs such as targeted therapies and immune checkpoint inhibitors (ICIs) have revolutionized the treatment of advanced melanoma. However, most prospective studies have excluded patients with melanoma brain metastasis (MBM) or included only patients with stable (steroid-free) or asymptomatic MBM. To assess the impact of novel treatments in patients with MBM in a real-world setting, we studied a cohort of consecutive patients over a period of sixteen years (2005 to 2021) in a large, tertiary referral center for melanoma (Erasmus MC, Rotterdam, the Netherlands). We found a significant improvement in overall survival in the modern (post-2015) era, associated with stereotactic radiotherapy and especially ICIs. These findings support that ICIs, if clinically feasible, should be considered first in clinical practice after a diagnosis of MBM.

**Abstract:**

Background: Melanoma brain metastasis (MBM) is associated with poor outcome, but targeted therapies (TTs) and immune checkpoint inhibitors (ICIs) have revolutionized treatment over the past decade. We assessed the impact of these treatments in a real-world setting. Methods: A single-center cohort study was performed at a large, tertiary referral center for melanoma (Erasmus MC, Rotterdam, the Netherlands). Overall survival (OS) was assessed before and after 2015, after which TTs and ICIs were increasingly prescribed. Results: There were 430 patients with MBM included; 152 pre-2015 and 278 post-2015. Median OS improved from 4.4 to 6.9 months (HR 0.67, *p* < 0.001) after 2015. TTs and ICIs prior to MBM diagnosis were associated with poorer median OS as compared to no prior systemic treatment (TTs: 2.0 vs. 10.9 and ICIs: 4.2 vs. 7.9 months, *p* < 0.001). ICIs directly after MBM diagnosis were associated with improved median OS as compared to no direct ICIs (21.5 vs. 4.2 months, *p* < 0.001). Stereotactic radiotherapy (SRT; HR 0.49, *p* = 0.013) and ICIs (HR 0.32, *p* < 0.001) were independently associated with improved OS. Conclusion: After 2015, OS significantly improved for patients with MBM, especially with SRT and ICIs. Demonstrating a large survival benefit, ICIs should be considered first after MBM diagnosis, if clinically feasible.

## 1. Introduction

Cutaneous melanoma has the highest risk of brain metastasis (MBM) of all solid tumors, spreading to the brain in over 50% of patients with metastatic disease [1,2,3]. Overall survival (OS) used to be <5 months, but several novel treatments have become available in the last decade, which have changed the perspectives of patients with MBM [2].

Whole brain radiotherapy (WBRT) and chemotherapy have limited efficacy in MBM, and WBRT is associated with cognitive impairment in the few long-term survivors [4,5,6]. These days, stereotactic radiotherapy (SRT) and especially targeted therapies (TTs; *BRAF-MEK* inhibitors) and immune checkpoint inhibitors (ICIs; anti-PD[L]1 and anti-CTLA4) have revolutionized treatment for patients with MBM [7,8,9,10,11,12,13,14]. Combined dabrafenib–trametinib showed an intracranial response rate (IRR) of 58%, but with limited duration of response (6.5 months) [7]. More durable responses are reported for ICIs, especially for combined nivolumab–ipilimumab. The ABC trial reported an IRR of 51% with a 5-year OS of 51% [8,9,10], and the CHECKMATE-204 trial had an IRR of 57% with a 1-year OS of 81.5% for this combination [11,15]. However, only a few phase II and III trials included limited numbers of patients with previously treated and/or symptomatic MBM, and these reported considerably lower IRRs (between 6% and 17% for ICIs) [8,11,15,16].

To assess the eligibility of individual patients for these novel treatments, the Melanoma-molGPA is often used in clinical practice as a prognostic scoring tool. This index is based on age, Karnofsky performance status (KPS), presence of extracranial metastasis (ECM), number of MBMs and, more recently, *BRAF*-mutation status [17,18,19]. However, since the index was derived from a retrospective cohort (*n* = 823) of patients between 2006 and 2015, its validity for modern cohorts is uncertain [17,20].

In the Netherlands, TTs and ICIs have been implemented in clinical practice for patients with MBM since their reimbursement almost a decade ago [21]. To assess the real-world impact of these treatments on MBM, we studied OS in a cohort of consecutive patients diagnosed with MBM over a period of sixteen years (2005 to 2021). Additionally, we examined the validity of the Melanoma-molGPA index for patients diagnosed with MBM in the modern treatment era.

## 2. Materials and Methods

### 2.1. Patient Selection

**Figure A1:**
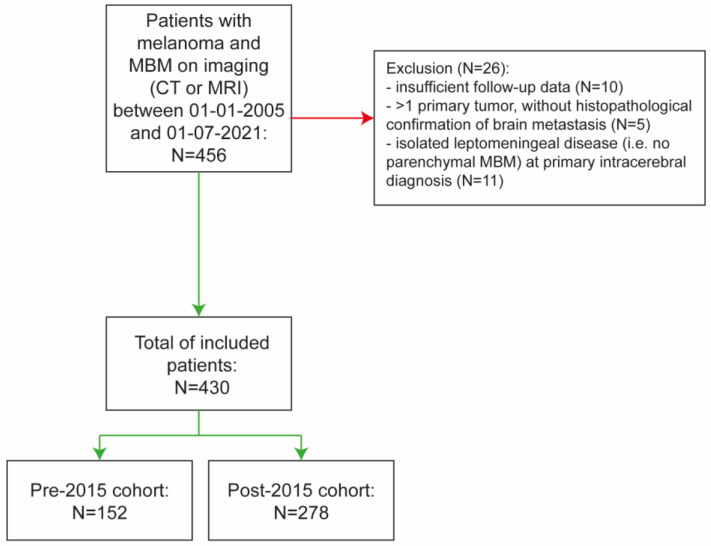
Flow chart of patient inclusion. Patients were subdivided into a pre-2015 cohort (diagnosis of melanoma brain metastasis (MBM) before 1 January 2015) and a post-2015 cohort (diagnosis of MBM after 1 January 2015). CT: computed tomography. MRI: magnetic resonance imaging.

This retrospective cohort study was performed at Erasmus MC Cancer Institute (Rotterdam, The Netherlands), a large, tertiary referral center for patients with melanoma. In this center, the treatment plan for patients with MBM is routinely discussed in a local multidisciplinary board which includes experienced medical oncologists, neuro-oncologists, radiotherapists, and neurosurgeons. We identified all consecutive patients with a diagnosis of cutaneous or mucosal melanoma and MBM referred to the Erasmus MC between 1 January 2005 and 1 July 2021 (Figure A1). The study was approved by the local Institutional Review Board (MEC-2020-0681).

### 2.2. Data Collection and Definitions

Two trained data managers (E.L.v.d.M., L.S.H.) retrieved data from the electronic patient records. All data were reassessed by two clinical physicians (S.H.A.E.D., J.L.M.J.). The following baseline characteristics were collected: age, sex, KPS, lactate dehydrogenase (LDH) level, *BRAF*-status, presence of symptoms of MBM (e.g., headache, nausea, epilepsy, focal deficits), number of MBMs, and status of ECM. Diagnosis of MBM was the date of first brain imaging (magnetic resonance imaging or computed tomography (CT)) that confirmed parenchymal MBM. Diagnosis of ECM was the date of first imaging (CT or 2-deoxy-2-[^18^F]fluoro-D-glucose-positron emission tomography-CT) of at least thorax and abdomen that confirmed metastasis (M1-disease). Melanoma brain metastasis was considered synchronous when diagnosed within one month of diagnosis of ECM and metachronous when diagnosed at least one month after diagnosis of ECM. The Melanoma-molGPA was calculated for each individual patient.

In addition, details of previous systemic treatments (i.e., given at any time prior to diagnosis of MBM) and treatments given directly after diagnosis of MBM (before the first physician assessed progression of MBM) were collected. Local treatments included surgical resection, SRT, and WBRT. Systemic treatments included chemotherapy (e.g., dacarbazine), TTs (e.g., vemurafenib, dabrafenib, encorafenib, alone or combined with cobimetinib, trametinib, binimetinib, respectively) and ICIs (e.g., pembrolizumab, atezolizumab, nivolumab, ipilimumab, or nivolumab–ipilimumab).

### 2.3. Statistical Analysis

**Figure A3:**
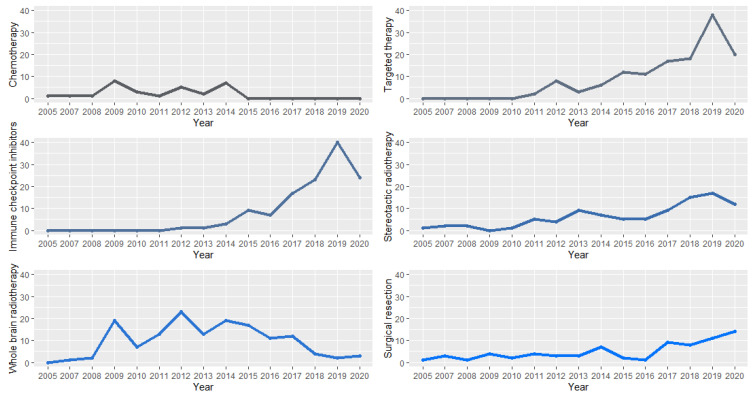
Number of started treatments each year at the Erasmus MC, at any time after diagnosis of melanoma brain metastasis.

Data cut-off was 4 March 2022. We created a total cohort and two consecutive time cohorts split 1 January 2015, as novel systemic treatments were increasingly prescribed after 2015 in the Netherlands (Table A1, Figure A3). The primary outcome was OS, defined as the time between diagnosis of MBM and death of any cause.

Continuous variables were described with medians (interquartile range (IQR)) and categorical variables with frequencies. The Mann–Whitney U test was used for continuous, and the Chi-squared test for categorical variables. For categorical variables with groups < 10 patients, the Fisher exact test was used. Missing data were omitted from analysis.

Kaplan–Meier (KM) and Cox Proportional Hazards (CPH) methods were used for time-to-event analysis and assessed with the Logrank and Likelihood ratio tests, respectively. For multivariate CPH modeling, backward elimination (i.e., including variables with *p* < 0.2 in univariate analysis) was applied, and interaction testing was performed. No correction for multiple testing was performed in these exploratory analyses. A two-sided *p*-value of 0.05 was taken as statistically significant. All analyses were performed using R version 4.0.2 (the R-Project, Auckland, New Zealand).

## 3. Results

### 3.1. Baseline Characteristics

We included 430 patients in total; 152 (35.3%) patients were categorized in the pre-2015 cohort and 278 (64.7%) in the post-2015 cohort (Figure A1). The majority of patients had a follow-up of at least 12 months (420 of 430 patients, 97.6%). Baseline characteristics at diagnosis of MBM are shown in Table 1. Overall, 133 (30.9%) patients had a KPS ≤ 70 and 321 of 430 (74.6%) patients had symptomatic MBM, with fewer symptomatic patients post-2015 (*n* = 193 of 278, 69.4%) as compared to pre-2015 (*n* = 128 of 152, 84.4%; *p* = 0.001).

### 3.2. Treatments over Time

Prior to the diagnosis of MBM, 104 of 430 (24.2%) patients had received one or more systemic treatments (Table 2). Fewer patients had received previous chemotherapy post-2015 compared to pre-2015 (1.4% vs. 6.6%; *p* = 0.008), whereas TTs and ICIs prior to MBM diagnosis had been administered more often after 2015 (3.3% to 13.3%, *p* < 0.001; 2.0% to 25.2%, *p* < 0.001, respectively).

Directly after diagnosis of MBM, 358 of 430 (83.3%) patients received one or more systemic and/or local treatments (Table 2). The frequency of SRT and surgical resection directly after diagnosis of MBM did not significantly change over time, whereas the frequency of WBRT and chemotherapy significantly decreased after 2015 (55.3% to 12.6%, *p* < 0.001; 14.5% to 1.4%, *p* < 0.001; respectively). The prescription of TTs and ICIs directly after diagnosis of MBM significantly increased after 2015 (10.5% to 33.8%, *p* < 0.001; 2.6% to 35.3%, *p* < 0.001; respectively).

### 3.3. Overall Survival

**Figure 1:**
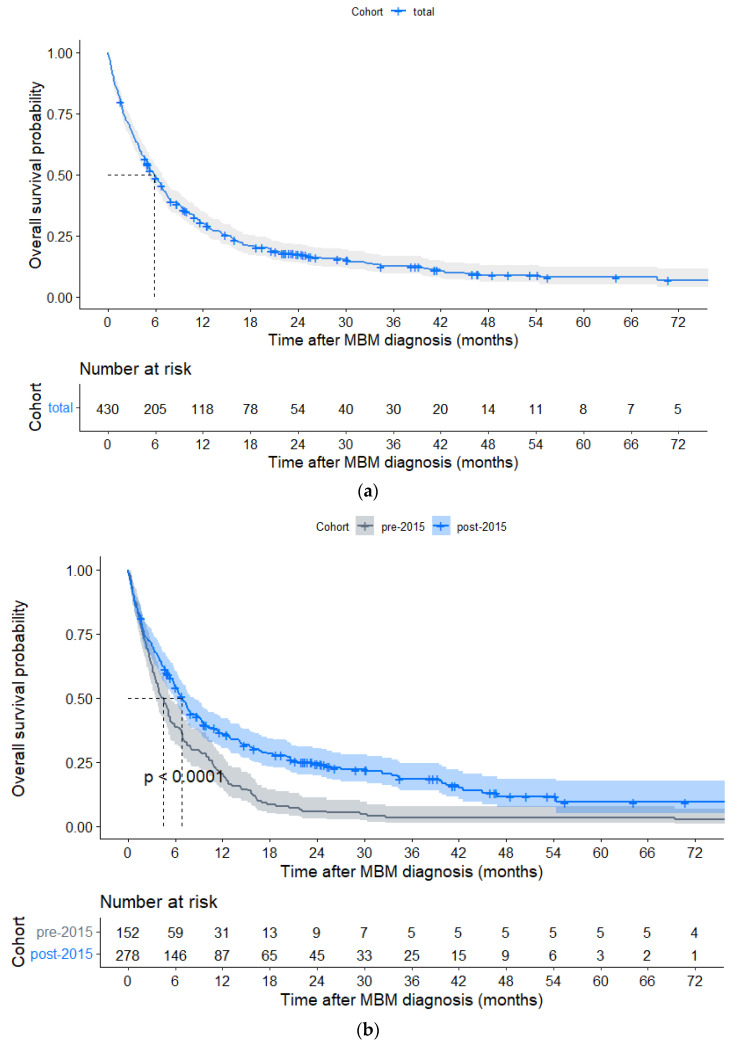
(**a**) Kaplan–Meier curve of overall survival in the total cohort (*n* = 430) of patients with melanoma brain metastasis (MBM). (**b**) Kaplan–Meier curves of overall survival of patients diagnosed with MBM pre-2015 (*n* = 152) and post-2015 (*n* = 278).

**Figure 2:**
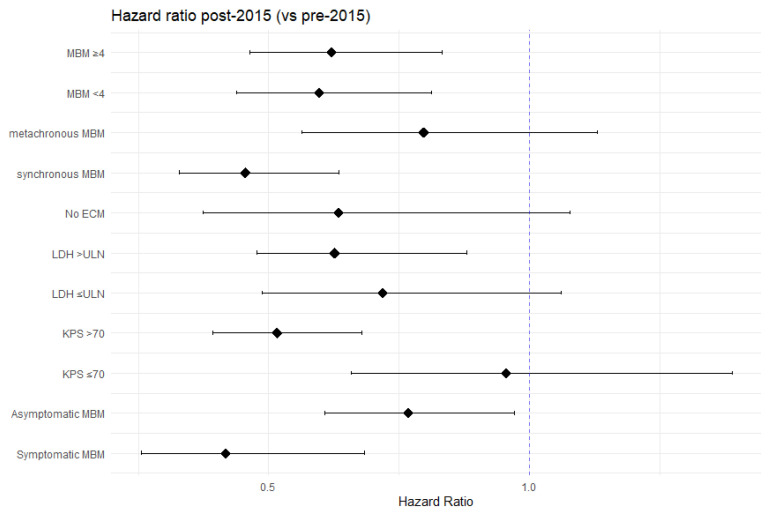
Forrest plot reflecting the hazard ratios (with 95% confidence interval) for patients diagnosed with melanoma brain metastasis (MBM) post-2015 (versus pre-2015) in several subgroups. Abbreviations: LDH: lactate dehydrogenase, ULN: upper limit of normal (247 U/L), KPS: Karnofsky performance status.

In the total cohort, median OS was 5.9 months (IQR 2.07–15.41), with 1- and 3-year OS rates of 30.2% and 12.5%, respectively (Figure 1a). Here, 21 of 430 (4.9%) patients had a survival time of at least 5 years since diagnosis of MBM, of whom 17 (81.0%) were diagnosed with MBM post-2015. At data cut-off, 65 of 430 (15.1%) patients, of whom 61 were diagnosed with MBM post-2015, were alive with a median follow-up of 23.8 (IQR 11.6–41.2) months. Median OS was significantly longer post-2015 as compared to pre-2015 (6.9 (IQR 2.07–23.39) vs. 4.4 (IQR 1.92–10.73) months, HR0.63, *p* < 0.001), especially in subgroups of patients with synchronous MBM, LDH levels > ULN and a KPS > 70 (Figure 1b and Figure 2, Table A2).

For patients receiving SRT and surgical resection directly after diagnosis of MBM, OS improved after 2015 (Table A3). Systemic treatments were not analyzed over time since their frequencies significantly changed over time (Table 2). To assess the impact of treatments in the modern era, we analyzed treatment subgroups in the post-2015 cohort (*n* = 278) only.

In these univariate analyses, a specific treatment before the diagnosis of MBM or directly after was compared to not having that specific treatment at that time. In addition, patients with symptomatic MBM were analyzed separately.

#### 3.3.1. Local Treatments Post-2015

**Figure 3:**
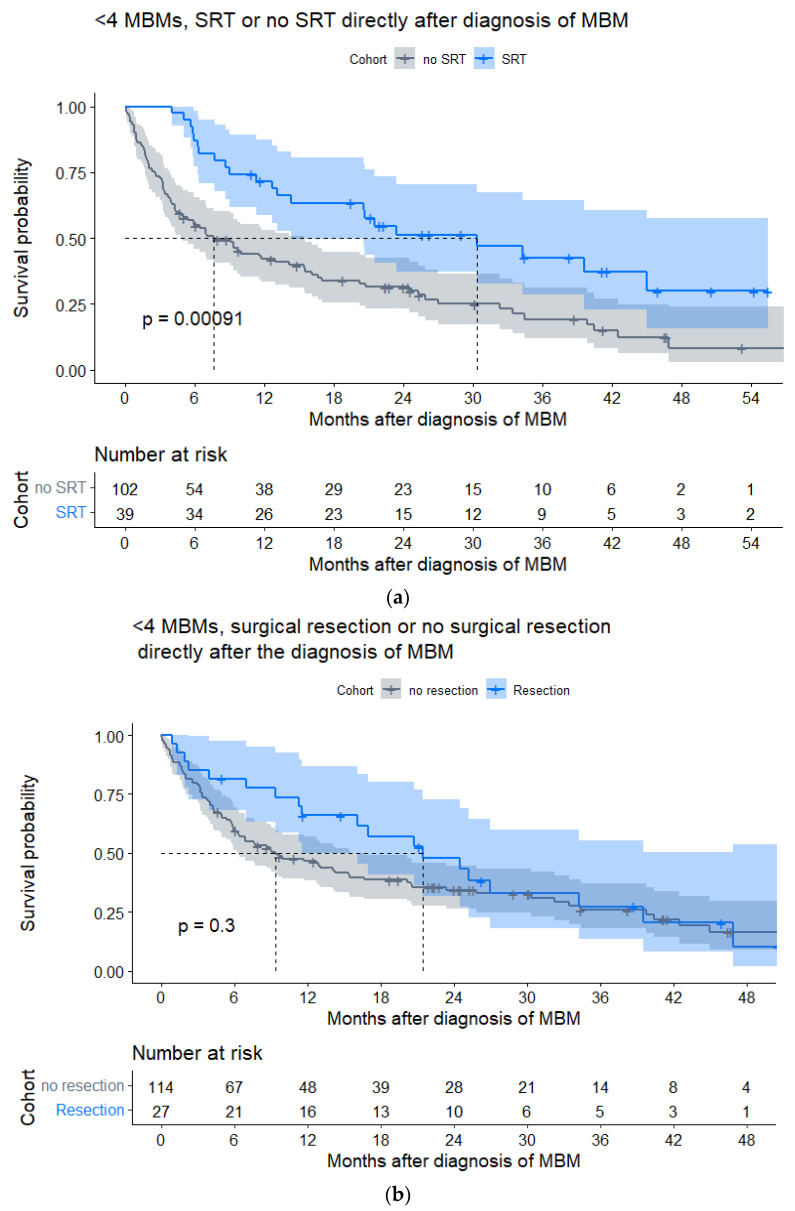
Kaplan–Meier estimates for local treatments in patients with melanoma brain metastasis (MBM) of the post-2015 cohort and with <4 MBMs (***n*** = 141). (**a**) Stereotactic radiotherapy (SRT) versus no stereotactic radiotherapy (no SRT) directly after diagnosis of MBM. (**b**) Surgical resection versus no surgical resection directly after diagnosis MBM.

**Figure A4:**
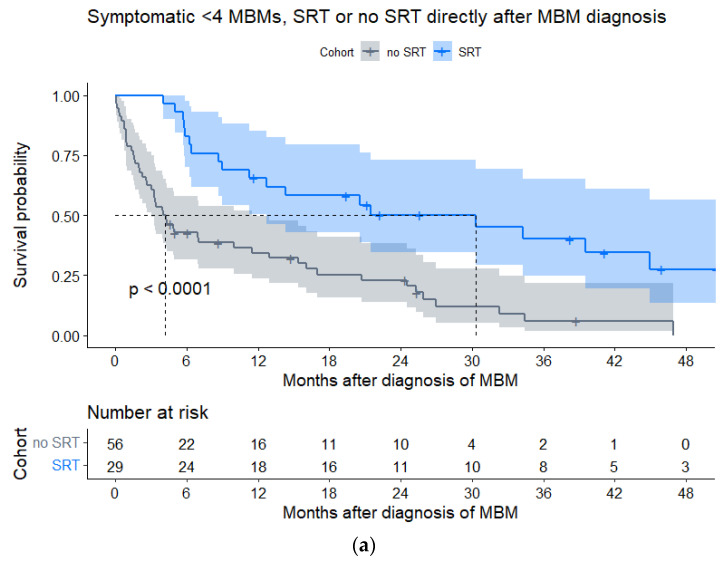

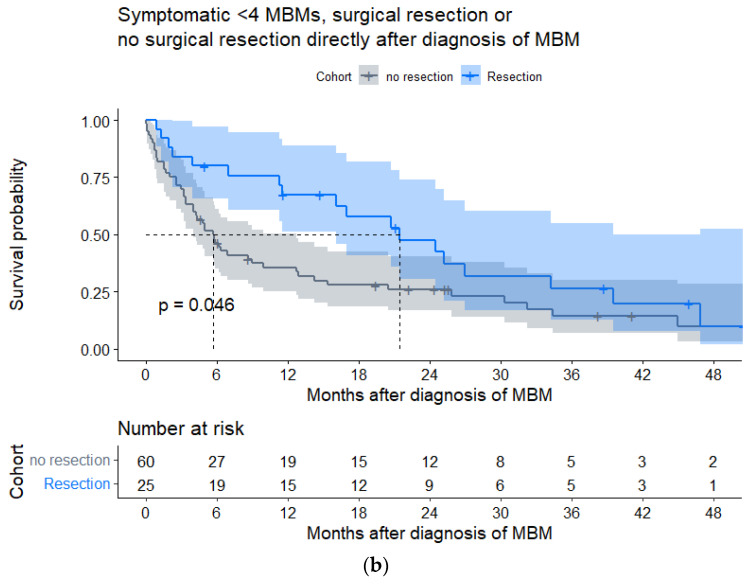
Kaplan–Meier curves in symptomatic patients with <4 melanoma brain metastases (MBMs) of the post-2015 cohort (*n* = 85) and local treatments given directly after diagnosis of MBM. (**a**) Stereotactic radiotherapy (SRT) or no SRT directly after MBM diagnosis. (**b**) Surgical resection or no surgical resection directly after MBM diagnosis.

Forty-two patients received SRT directly after diagnosis of MBM, of whom 39 (92.9%) had <4 MBMs. In these patients, SRT significantly improved OS as compared to patients without SRT (median OS 30.3 (IQR 9.0–NA) vs. 7.6 (IQR 2.6–32.3) months, HR0.46, *p* < 0.001; Figure 3a, Table A4). In patients with <4 MBMs and symptomatic MBM, SRT remained associated with improved OS (Figure A4a).

In patients with <4 MBMs and symptomatic MBM, surgical resection directly after diagnosis of MBM was associated with improved OS as compared to no surgical resection (median OS 21.5 (IQR 11.3–29.6) vs. 5.7 (IQR 2.3–25.9) months, HR0.58, *p* = 0.046; Figure A4b).

#### 3.3.2. Systemic Treatments Post-2015

**Figure 4:**
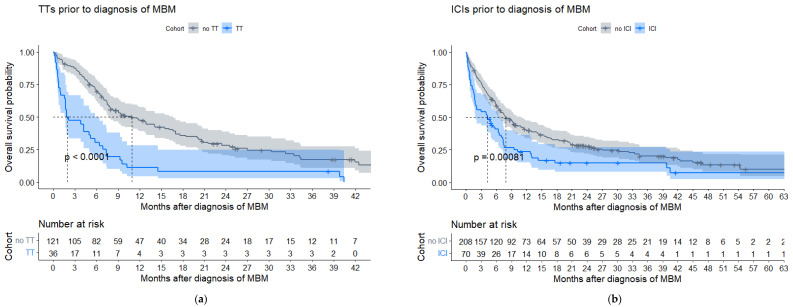

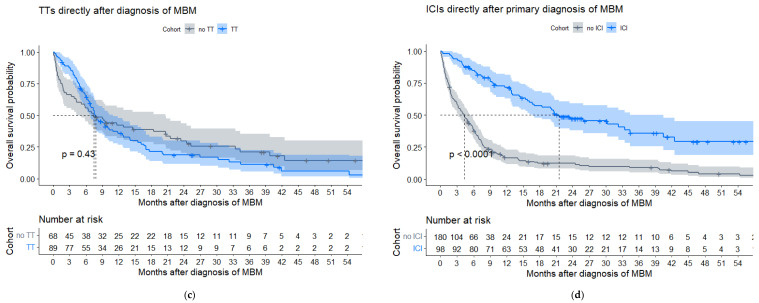
Kaplan–Meier estimates of systemic treatments given to patients with melanoma brain metastasis (MBM) of the post-2015 cohort (*n* = 278). (**a**) TTs or no TTs prior to diagnosis of MBM in patients with a targetable *BRAF* V600E or K+ mutation (*n* = 157). (**b**) ICIs or no ICIs prior to diagnosis of MBM. (**c**) TTs or no TTs directly after diagnosis of MBM in patients with a *BRAF* V600E or K+ mutation (*n* = 157). (**d**) ICIs or no ICIs directly after diagnosis of MBM.

**Figure A5:**
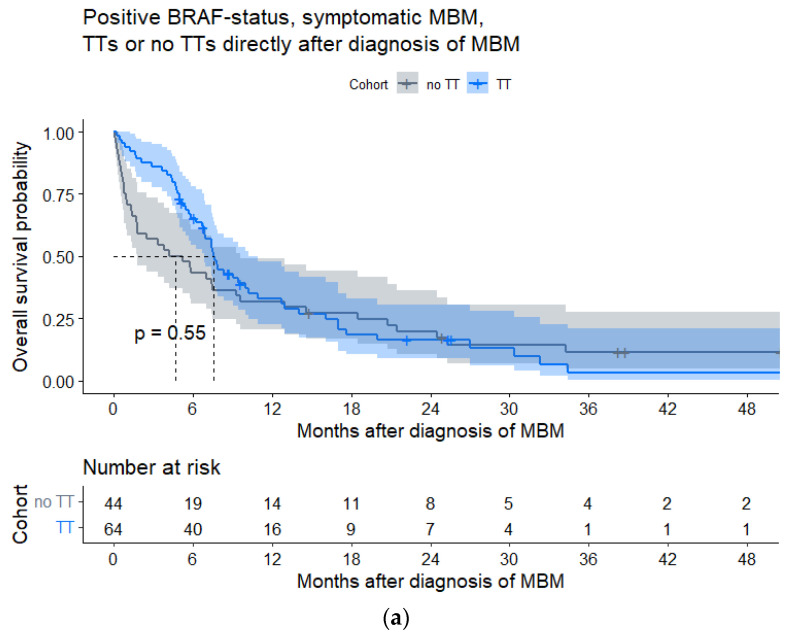

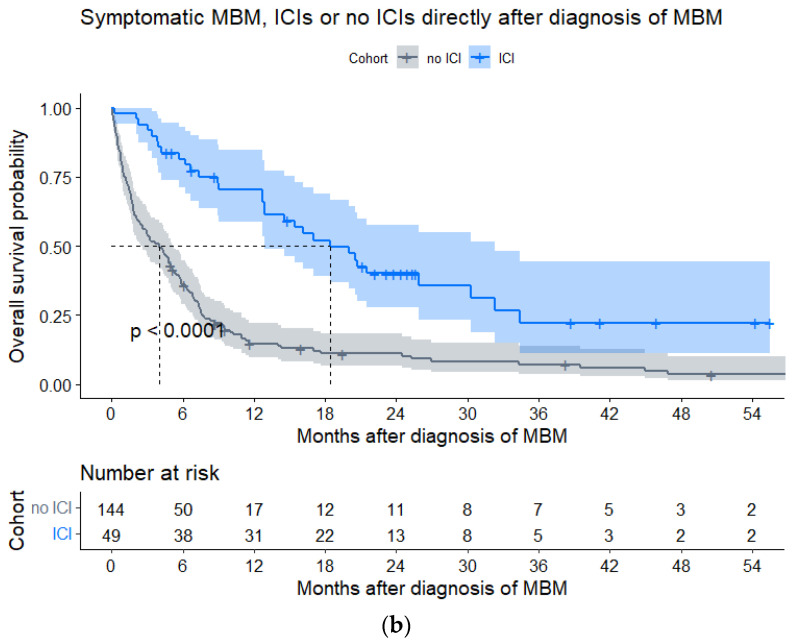
Kaplan–Meier curves in symptomatic patients with melanoma brain metastasis (MBM) in the post-2015 cohort (*n* = 193) and systemic treatments given directly after diagnosis of MBM. (**a**) targeted therapies (TTs) or no TTs directly after diagnosis of MBM in patients with a positive *BRAF* V600E+/K-status (*n* = 108). (**b**) immune checkpoint inhibitors (ICIs) or no ICIs directly after diagnosis of MBM.

In patients with *BRAF* V600E+/K-mutated melanoma (*n* = 157), TTs prior to the diagnosis of MBM were associated with a shorter median OS as compared to no prior TTs (2.0 (IQR 0.8–7.1) vs. 10.9 (IQR 5.2–27.0) months, HR2.67, *p* < 0.001; Figure 4a, Table A4). In patients with prior TTs, 58.3% of patients had also been previously treated with ICIs (Table A5a). Of all patients with TTs directly after diagnosis of MBM (*n* = 89), 64 (71.9%) had symptomatic MBM (Figure A5a). No significantly different OS was found between patients with and without TTs directly after MBM diagnosis (7.8 (IQR 4.8–17.0) vs. 7.4 (IQR 1.5–33.4) months, HR1.15, *p* = 0.43; Figure 4c, Table A4).

Patients treated with ICIs prior to the diagnosis of MBM had a poorer median OS as compared to patients without prior ICIs (4.2 (IQR 1.0–10.2) vs. 7.9 (IQR 3.1–27.0) months, HR 1.67, *p* < 0.001) and 30.0% of patients with prior ICIs had also been previously treated with TTs (Figure 4b, Table A4 and Table A5b). Patients with ICIs directly after diagnosis of MBM had a better median OS as compared to patients without ICIs directly after MBM (21.5 (IQR 9.4–NA) vs. 4.2 (IQR 1.4–8.0) months, HR 0.28, *p* < 0.001; Figure 4d, Table A4), which was also confirmed in a subgroup of symptomatic patients (18.5 (IQR 9.0–34.4) vs. 4.0 (IQR 1.1–7.6) months, HR 0.33, *p* < 0.001; Figure A5b). Of all patients (*n* = 98) with ICIs directly after MBM diagnosis, 44 (44.9%) were alive at data cut-off, and 17 (17.3%) patients had a survival time of at least 5 years after diagnosis of MBM. In 23 of 98 (23.5%) patients, ICIs were combined with SRT.

### 3.4. Independent Prognostic Variables

In multivariate analysis of the post-2015 cohort (*n* = 278), symptomatic MBM (HR 1.74 [1.21–2.50], *p* = 0.003) and metachronous MBM (HR 2.73 [1.50–4.95], *p* < 0.001) were independently associated with poorer OS, whereas a KPS of >70 (HR 0.51 [0.37–0.70], *p* < 0.001), SRT (HR 0.49 [0.28–0.86], *p* = 0.013) and ICIs directly after diagnosis of MBM (HR 0.32 [0.22–0.47], *p* < 0.001) were independently associated with an improved OS (Table 3).

### 3.5. Melanoma-molGPA

**Figure 5:**
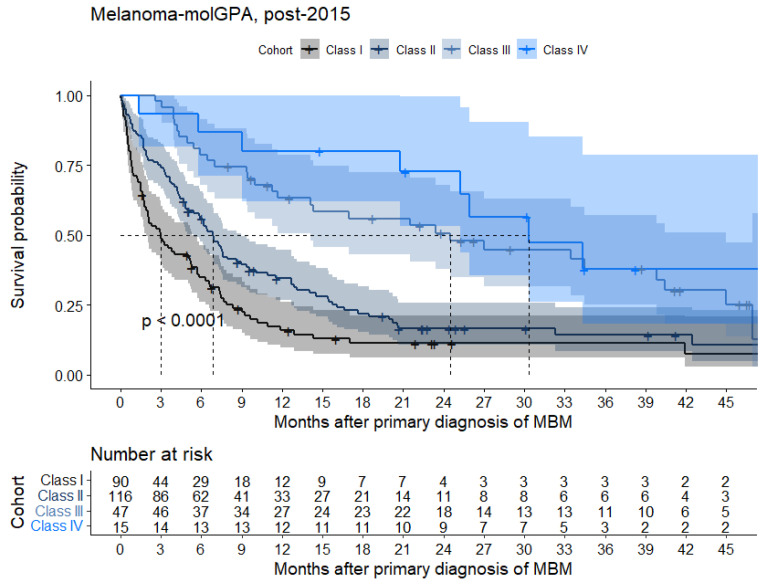
Kaplan–Meier estimates for overall survival of patients diagnosed with melanoma brain metastases (MBM) in the post-2015 cohort of patients with available model input parameters (*n* = 268) according to the different prognostic classes (I to IV) of melanoma-molGPA score.

The Melanoma-molGPA could be assessed for 268 of 278 (96.4%) patients post-2015. The melanoma-molGPA subclasses (I, II, III, and IV, respectively) showed a subsequent improvement of OS (median OS 3.0, 6.9, 24.5, and 30.3 months, respectively, *p* < 0.001; Figure 5, Table A6).

### 3.6. Switching from Targeted Therapy to Immune Checkpoint Inhibition

Here, 18 of 430 (4.2%) patients initiated with TTs directly after diagnosis of MBM and switched to ICIs when their performance status improved and/or imaging showed tumor response at a median time of 6.1 months (IQR 3.9–7.0) after the start of TTs (Table A7). Three of eighteen (16.7%) patients remained stable since the switch to ICIs and were alive at data cut-off, with a median survival time of 25.5 months (IQR 21.7–27.2) since ICI initiation. Three of eighteen (16.7%) patients had progressive disease after ICI initiation but received no further treatment due to poor clinical condition, with a median OS of 11.9 months (IQR 7.6–16.3) since ICI initiation. Twelve of eighteen (66.7%) patients had progressive disease on ICIs and switched back to TTs, with a median OS of 11.6 months (IQR 6.5–25.5) since ICI initiation.

## 4. Discussion

Since 2015, novel systemic therapies (TTs and ICIs) and SRT have replaced chemotherapy and WBRT for patients with MBM, resulting in a significantly improved OS. Compared to clinical trials, real-world patients with MBM usually have a worse clinical condition, which is illustrated by the high number of patients with poor KPS and symptomatic MBM in this cohort.

Surgical resection and SRT remained important treatment options after 2015, although their frequency directly after diagnosis of MBM did not significantly increase over time. Especially SRT was associated with a beneficial impact on survival (median OS 30.5 months) and remained independently associated with improved OS. Although the efficacy of SRT has been demonstrated in patients with up to 10 MBMs [22,23], SRT was almost exclusively reserved for patients with <4 MBMs in the current cohort.

The increased use of TTs (10.5% to 33.8%) and ICIs (2.6% to 35.3%) post-2015, directly after diagnosis of MBM, reflects the clinical approval of different novel drugs by the European Medicines Agency (EMA) and their reimbursement in the Netherlands since 2015 [24]. Bander et al. showed an even higher use of ICIs (77%) in their American cohort, likely resulting from the earlier approval of these drugs by the Food and Drug Administration (FDA) and from differences in therapeutic approaches between centers [25,26].

Patients who received TTs or ICIs to treat ECM, before the diagnosis of MBM, had poor survival (median OS of 2.0 and 4.2 months, respectively) as they had secondary resistance with intracranial disease progression on/after these treatments. Other real-world studies confirm the poor OS of patients with systemic treatments prior to the diagnosis of MBM [26,27]. Most importantly, a significantly favorable OS was seen with ICIs directly after the diagnosis of MBM (median OS 21.5 months), even in symptomatic patients (18.5 months). Although median follow-up is limited, the tail of the OS curve (Figure 4d) showed a considerable group of patients with long-term survival, as illustrated by >17% of patients with ICIs who lived >5 years after diagnosis of MBM. This confirms in a real-world setting the efficacy of ICIs in MBM, as reported by the benchmark phase II and III trials [10,15]. Therefore, ICIs should be considered first after diagnosis of MBM, if clinically feasible.

The Melanoma-molGPA, based on an MBM cohort between 2006 and 2015, remained a valid prognostic tool in patients diagnosed with MBM between 2015 and 2021 [17,18]. Therefore, clinicians may continue to use this tool to predict prognosis and assess the eligibility of individual patients for specific treatments.

Since TTs can induce rapid tumor responses, these drugs were frequently administered (>70%) to patients with symptomatic MBM and a poor performance status. However, TTs lack durable responses and it was recently demonstrated that first-line nivolumab–ipilimumab followed by *BRAF/MEK*-inhibition on progression had the most favorable OS in metastatic melanoma [28]. Nevertheless, in patients with a *BRAF* V600E+/K-mutation and an initial poor performance status, induction with TTs provides an opportunity for effective ICI treatment after performance has improved [29]. Although our study was not designed to compare treatment strategies, we assessed the outcome of 18 patients who were first treated with induction TTs after the diagnosis of MBM, followed by ICIs. Only three (16.7%) patients benefitted from the switch to ICIs, with stable disease and a median OS of 25.5 months since ICIs. Although (pre-)clinical studies have shown that an immune-resistant phenotype might arise after progression on *BRAF/MEK*-inhibition, it is unknown whether the switch to ICIs before progression on TTs could be effective, and it is worthwhile to further investigate this prospectively [8,30].

This study was designed to assess the potential survival gain in the brain for patients with MBM in a real-world setting after the introduction of novel systemic treatments. The retrospective design is a limitation, as is the lack of information on steroid use. The inclusion of a single center might provide additional bias. However, this tertiary center receives referrals from a large region in the Netherlands, and each consecutive patient is treated and followed in this center until end-of-life care or death. Therefore, this data set provides detailed information over a large period of time, fully capturing the changing treatment landscape.

## 5. Conclusions

Overall survival has improved for patients with MBM after 2015 and is associated with the use of SRT and ICIs directly after diagnosis of MBM. Immune checkpoint inhibitors (ICIs) showed an important survival benefit and should be considered first after the diagnosis of MBM, if clinically feasible. The Melanoma-molGPA remains a valid prognostic tool for clinicians in the modern treatment era for patients with MBM. To further improve the prognosis of patients with MBM, future research should focus on optimizing treatment sequencing, such as switching from TTs to ICIs, especially in patients with an initial poor performance.

## Figures and Tables

**Table 1:** Baseline characteristics of patients at diagnosis of melanoma brain metastasis (MBM).

	**Patients (%)**			
	Total	Pre-2015	Post-2015	*p*-Value ^1^
Variable	430	152	278	
Age at diagnosis of MBM, years				*0.005*
Median (interquartile range)	63.1 (52.3–71.7)	60.6 (49.2–69.1)	64.5 (53.4–72.9)	
Sex				0.192
Women	173 (40.2)	68 (44.7)	105 (37.8)	
Men	257 (59.8)	84 (55.3)	173 (62.2)	
Karnofsky performance status (KPS)				0.722
≤70	133 (30.9)	43 (28.3)	90 (32.4)	
80	141 (32.8)	51 (33.6)	90 (32.4)	
90–100	131 (30.5)	42 (27.6)	89 (32.0)	
Unknown ^2^	25 (5.8)	16 (10.5)	9 (3.2)	
LDH at MBM diagnosis				0.136
≤ULN	162 (37.7)	40 (26.3)	122 (43.9)	
>ULN	168 (39.1)	55 (36.2)	113 (47.8)	
Unknown ^2^	100 (23.3)	57 (37.5)	43 (15.5)	
*BRAF* mutational status				*0.013*
Wildtype	145 (33.7)	45 (29.6)	100 (36.0)	
V600E+ or K	192 (44.7)	35 (23.0)	157 (56.5)	
Other	15 (3.5)	2 (1.3)	13 (4.7)	
Unknown ^2^	78 (18.1)	70 (46.1)	8 (2.9)	
Time between first diagnosis of melanoma and diagnosis of MBM, months				
Median (interquartile range)	37.0 (11.0–68.0)	36.5 (12.0–59.3)	37.0 (10.0–70.0)	0.720
Time between first diagnosis of ECM and diagnosis of MBM, months ^3^				
Median (interquartile range)	8.0 (5.0–17.0)	9.0 (4.0–15.0)	8.0 (5.0–19.0)	0.40
Symptomatic MBM				*0.001*
Yes	321 (74.6)	128 (84.2)	193 (69.4)	
No	109 (25.3)	24 (15.8)	85 (30.6)	
Number of MBMs				0.533
1	123 (28.6)	38 (25.0)	86 (30.9)	
2	57 (13.3)	23 (15.1)	34 (12.2)	
3	35 (8.1)	14 (9.2)	21 (7.6)	
≥4	215 (50.0)	77 (50.7)	137 (49.3)	
Status of ECM				0.391
No ECM	75 (17.4)	31 (20.4)	44 (15.8)	
MBM synchronous with ECM	187 (43.5)	65 (42.8)	122 (43.9)	
MBM metachronous with ECM	153 (35.6)	49 (32.2)	104 (37.4)	
Unknown ^2^	15 (3.5)	7 (4.6)	8 (2.9)	

^1^*p*-values are given for the comparison between pre- and post-2015 time cohorts; significant *p*-values (<0.05) are italic. ^2^ The “unknown” category was omitted from statistical testing. ^3^ Analysed in patients with metachronous MBM (*n* = 153). Abbreviations: LDH: lactate dehydrogenase, ULN: upper limit of normal (247 U/L), ECM: extracranial metastasis.

**Table 2:** Treatment patterns before and directly after diagnosis of melanoma brain metastasis (MBM).

	Patients (%)			
	Total	Pre-2015	Post-2015	*p*-Value ^1^
Variable	430	152	278	
**Treatments prior to MBM diagnosis**				
***Systemic treatments***				
**Chemotherapy**				*0.008*
Yes	14 (3.3)	10 (6.6)	4 (1.4)	
No	416 (96.7)	142 (93.4)	274 (98.6)	
**Targeted therapy**				*<0.001*
Yes	42 (9.8)	5 (3.3)	37 (13.3)	
No	388 (90.2)	147 (96.7)	241 (86.7)	
**Immune checkpoint inhibition**				*<0.001*
Yes	73 (17.0)	3 (2.0)	70 (25.2)	
No	357 (83.0)	149 (98.0)	208 (74.8)	
**Treatments directly after MBM diagnosis**				
*Local treatments*				
SRT				0.684
Yes	62 (14.4)	20 (13.2)	42 (15.1)	
No	368 (85.6)	132 (86.8)	236 (84.9)	
WBRT				
Yes	120 (27.9)	84 (55.3)	35 (12.6)	*<0.001*
No	310 (72.1)	68 (44.7)	243 (87.4)	
**Surgical resection**				0.203
Yes	52 (12.1)	23 (15.1)	29 (10.4)	
No	378 (87.9)	129 (84.9)	249 (89.6)	
*Systemic treatments*				
**Chemotherapy**				*<0.001*
Yes	22 (5.1)	22 (14.5)	4 (1.4)	
No	408 (94.9)	130 (85.5)	274 (98.6)	
**Targeted therapy**				*<0.001*
Yes	110 (25.6)	16 (10.5)	94 (33.8)	
No	320 (74.4)	136 (89.5)	184 (66.2)	
**Immune checkpoint inhibition**				*<0.001*
Yes	101 (23.5)	4 (2.6)	98 (35.3)	
No	329 (76.5)	148 (97.4)	180 (64.7)	
**Best supportive care**				0.726
Yes	73 (17.0)	24 (15.8)	49 (17.6)	
No	357 (83.0)	128 (84.2)	229 (82.4)	

^1^*p*-Values were calculated to compare pre- and post-2015 time cohorts; significant *p*-values (<0.05) are in italic. Abbreviations: SRT: stereotactic radiotherapy, WBRT: whole brain radiotherapy.

**Table 3:** Cox proportional hazards model for overall survival of patients diagnosed with melanoma brain metastasis (MBM) in the post-2015 cohort (*n* = 278).

	Univariate Analysis		Multivariate Analysis	
Variable	HR [95%CI]	*p*-Value	HR [95%CI]	*p*-Value
Age at MBM diagnosis	1.012 [1.002–1.023]	0.017	-	-
Time between primary diagnosis melanoma and MBM	1.001 [0.999–1.003]	0.244	-	-
Female sex (ref. male)	0.945 [0.717–1.245]	0.686	-	-
LDH > ULN (ref. ≤ ULN)	1.701 [1.269–2.279]	<0.001	1.305 [0.941–1.808]	0.110
KPS > 70 (ref. ≤ 70)	0.331 [0.250–0.440]	<0.001	0.511 [0.371–0.703]	*<0.001*
≥4 MBMs (ref. 1–3)	1.983 [1.509–2.606]	<0.001	-	-
*BRAF* V600E+/K mutation (ref. wildtype)	0.913 [0.686–1.215]	0.546	-	-
Symptomatic MBM (ref. no)	1.918 [1.410–2.609]	<0.001	1.741 [1.210–2.504]	*0.003*
MBM synchronous with ECM (ref. no ECM)	1.499 [0.978–2.298]	0.063	1.412 [0.790–2.524]	0.244
MBM metachronous to ECM (ref. no ECM)	2.815 [1.830–4.330]	<0.001	2.726 [1.501–4.951]	*<0.001*
Previous TTs (ref. no)	2.247 [1.565–3.225]	<0.001	-	-
Previous ICIs (ref. no)	1.665 [1.232–2.250]	<0.001	0.687 [0.456–1.035]	0.072
TTs after MBM diagnosis (ref. no)	1.062 [0.803–1.404]	0.673	-	-
ICIs after MBM diagnosis (ref. no)	0.280 [0.205–0.384]	<0.001	0.323 [0.221–0.472]	*<0.001*
Surgical resection (ref. no)	0.566 [0.357–0.899]	0.009	0.656 [0.348–1.237]	0.192
SRT (ref. no)	0.412 [0.271–0.626]	<0.001	0.493 [0.283–0.860]	*0.013*

Significant *p*-values (<0.05) in multivariate analysis are in italic. Abbreviations: LDH: lactate dehydrogenase, ULN: upper limit of normal (247 U/L), KPS: Karnofsky performance status, ECM: extracranial metastasis, TTs: targeted therapies, ICIs: immune checkpoint inhibitors, SRT: stereotactic radiotherapy.

**Figure A2:**
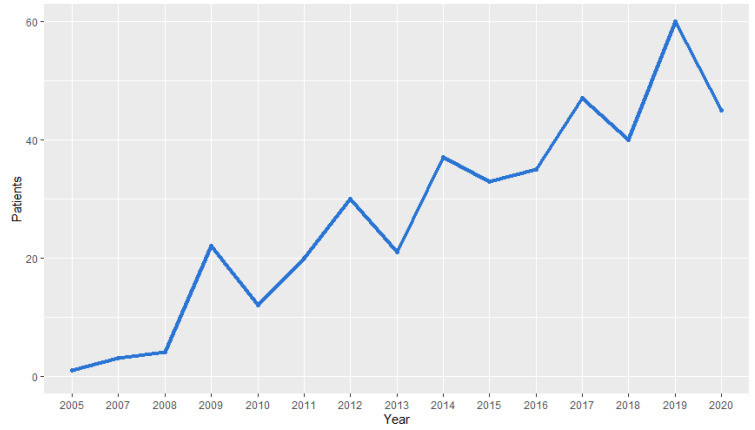
Number of patients referred each year to the Erasmus MC with newly diagnosed melanoma brain metastasis.

**Figure A6:**
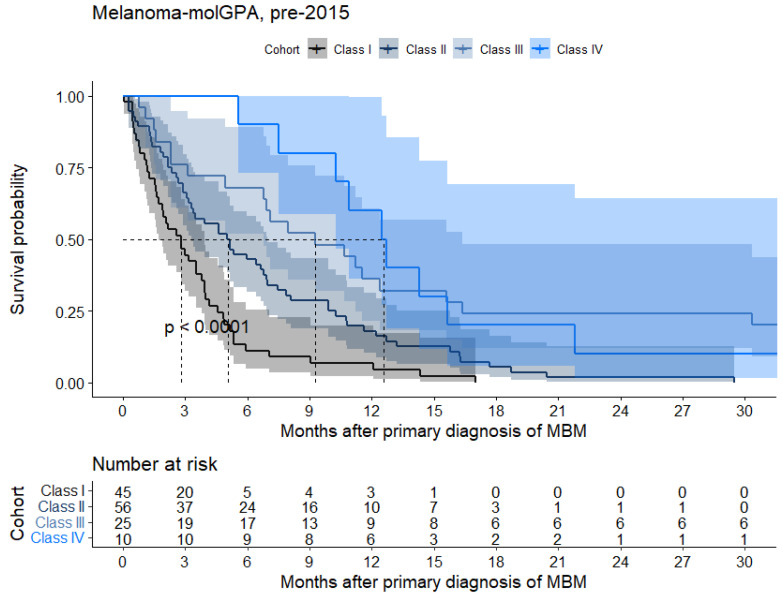
Kaplan–Meier estimates for overall survival in patients with melanoma brain metastasis (MBM) in the pre-2015 cohort of patients with available model input parameters (*n* = 136) according to the different prognostic classes (I to IV) of melanoma-molGPA score.

## Data Availability

The data presented in this study are available within the article or as Appendix A.

## Appendix A

**Table A1:** Yearly incidence of patients with newly diagnosed melanoma brain metastasis (MBM) referred to the Erasmus MC, and yearly treatment prescriptions (at any time after MBM diagnosis).

Year of Prescription	No. of Patients	Chemo-therapy	Targeted Therapy	Immune Checkpoint Inhibition	Stereotactic Radiotherapy	Whole Brain Radiotherapy	Surgical Resection
2005	1	1	0	0	1	0	1
2007	3	1	0	0	2	1	3
2008	4	1	0	0	2	2	1
2009	22	8	0	0	0	19	4
2010	12	3	0	0	1	7	2
2011	20	1	2	0	5	13	4
2012	30	5	8	1	4	23	3
2013	21	2	3	1	9	13	3
2014	37	7	6	3	7	19	7
2015	33	0	12	9	5	17	2
2016	35	0	11	7	5	11	1
2017	47	0	17	17	9	12	9
2018	40	0	18	23	15	4	8
2019	60	0	38	40	17	2	11
2020	45	0	20	24	12	3	14
2021 *	20	0	16	13	6	1	3

* Only the first 6 months of 2021 were included.

**Table A2:** Overall survival for subgroups of patients diagnosed with melanoma brain metastasis (MBM) in the total cohort (*n* = 430), pre-2015 cohort (*n* = 152) and post-2015 cohort (*n* = 278). Significance testing was performed to compare the pre-2015 and post-2015 cohorts, with significant *p*-values (<0.05) in italic.

	Total Cohort	Pre-2015 Cohort	Post-2015 Cohort	HR [95%CI] (Ref. = Pre-2015 Cohort)	*p*-Value between Time Cohorts
Variables	Median OS (Months)	Median OS (Months)	Median OS (Months)		
	5.88	4.44	6.87	0.626 [0.507–0.773]	*<0.001*
Symptoms of MBM					
Asymptomatic	10.05	4.57	14.13	0.418 [0.255–0.684]	*<0.001*
Symptomatic	5.03	4.37	5.68	0.768 [0.607–0.971]	*0.027*
KPS					
≤70	2.07	2.60	1.81	0.956 [0.659–1.389]	0.816
>70	9.04	6.77	11.30	0.516 [0.392–0.679]	*<0.001*
LDH level					
≤ULN	9.36	7.00	10.05	0.720 [0.488–1.061]	0.097
>ULN	3.55	2.73	4.67	0.627 [0.447–0.880]	*0.007*
ECM status					
None	11.53	11.20	20.73	0.635 [0.374–1.078]	0.092
Synchronous MBM	6.44	4.17	8.97	0.456 [0.329–0.634]	*<0.001*
Metachronous MBM	4.01	3.55	4.22	0.798 [0.563–1.130]	0.210
Number of MBMs					
<4	9.66	6.93	12.71	0.597 [0.439–0.813]	*0.001*
≥4	3.94	2.99	5.19	0.621 [0.464–0.832]	*0.001*

Abbreviations: KPS: Karnofsky performance status, LDH: lactate dehydrogenase, ULN: upper limit of normal (247 U/L), ECM: extracranial metastasis.

**Table A3:** Overall survival (OS) for local treatment directly after MBM diagnosis, in patients with <4 melanoma brain metastases (MBMs) in the total cohort (*n* = 216), pre-2015 cohort (*n* = 75) and post-2015 cohort (*n* = 141).

	Total Cohort	Pre-2015 Cohort	Post-2015 Cohort	HR [95%CI] (Ref. = Pre-2015 Cohort)	*p*-Value between Time Cohorts
	Median OS (Months)	Median OS (Months)	Median OS (Months)		
*Local treatments, <4 MBMs (n = 216)*					
SRT					
Yes	20.4	12.8	30.3	0.515 [0.272–0.974]	*0.04*
No	6.8	6.0	7.6	0.620 [0.436–0.883]	*0.007*
Surgical resection					
Yes	11.5	9.9	21.5	0.470 [0.249–0.890]	*0.02*
No	7.6	6.5	9.4	0.617 [0.432–0.882]	*0.007*

Abbreviations: SRT: stereotactic radiotherapy.

**Table A4:** Overall survival (OS) for treatment subgroups of patients with melanoma brain metastasis (MBM) in the post-2015 cohort (*n* = 278) and for local treatments in the post-2015 cohort of patients with <4 MBMs (*n* = 141).

	Median OS (Months)	1-Year Probability OS (%)	HR [95%CI]	*p*-Value
**Treatments prior to diagnosis of MBM**				
*Systemic treatments (n = 278)*				
No prior TTs ^1^	10.94	0.47	ref.	
Prior TTs ^1^	1.95	0.11	2.670 [1.793–3.974]	*<0.001*
No prior ICIs	7.89	0.40	ref.	
Prior ICIs	4.24	0.24	1.665 [1.232–2.250]	*<0.001*
**Treatments directly after diagnosis of MBM**				
*Local treatments, <4 MBMs (n = 141)*				
No SRT	7.62	0.42	ref.	
SRT	30.33	0.72	0.456 [0.284–0.734]	*<0.001*
No surgical resection	9.36	0.47	ref.	
Surgical resection	21.49	0.66	0.769 [0.466–1.268]	0.303
*Systemic treatments (n = 278)*				
No TTs ^1^	7.41	0.42	ref.	
TTs ^1^	7.79	0.36	1.154 [0.804–1.653]	0.435
No ICIs	4.24	0.16	ref.	
ICIs	21.49	0.72	0.280 [0.205–0.384]	*<0.001*

^1^ In patients with a targetable *BRAF*-mutation (*n* = 157). Abbreviations: TTs: targeted therapies, ICIs: immune checkpoint inhibitors, SRT: stereotactic radiotherapy.

**Table A5:** (**a**) Baseline characteristics of patients with a *BRAF* V600E+/K mutated melanoma and melanoma brain metastasis (MBM) from the post-2015 cohort (*n* = 157) treated with and without targeted therapies (TTs) prior to MBM diagnosis. (**b**) Baseline characteristics of patients from the post-2015 cohort (*n* = 278) treated with or without immune checkpoint inhibitors (ICIs) prior to MBM diagnosis.

(a)			
Variables	Previous TTs (%)	No Previous TTs (%)	*p*-Value
	36 (100)	121 (100)	
Number of MBMs			*0.003*
1–3	7 (19.4)	57 (47.1)	
≥4	29 (80.6)	64 (52.9)	
ECM status			*<0.001*
None	1 (2.8)	19 (15.7)	
Synchronous MBM	1 (2.8)	69 (57.0)	
Metachronous MBM	33 (91.7)	31 (25.6)	
Unknown ^1^	1 (2.8)	2 (1.7)	
LDH status			0.099
≤ULN	15 (41.7)	57 (47.1)	
>ULN	20 (55.6)	48 (39.7)	
Unknown ^1^	1 (2.8)	16 (13.2)	
Symptomatic MBM			*0.040*
No	6 (16.7)	43 (35.5)	
Yes	30 (83.3)	78 (64.5)	
Previous ICIs			*<0.001*
No	15 (41.7)	103 (85.1)	
Yes	21 (58.3)	18 (14.9)	
First line TTs			*<0.001*
No	28 (77.8)	40 (33.1)	
Yes	8 (22.2)	81 (66.9)	
First line ICIs			*0.013*
No	31 (86.1)	77 (63.6)	
Yes	5 (13.9)	44 (36.4)	
Median time between primary diagnosis of ECM and MBM (months) ^2^	8.0 IQR (5.0–19.0)	7.0 IQR (4.0–10.0)	0.40
(**b**)			
**Variables**	**Previous ICI (%)**	**No previous ICI (%)**	***p*-Value**
	70 (100)	208 (100)	
Number of MBMs			0.10
1–3	29 (41.4)	112 (53.8)	
≥4	41 (58.6)	96 (46.2)	
ECM status			*<0.001*
None	4 (5.7)	40 (19.2)	
Synchronous MBM	6 (8.6)	116 (55.8)	
Metachronous MBM	58 (82.9)	46 (22.1)	
Unknown ^1^	2 (2.8)	6 (2.9)	
LDH status			*0.018*
≤ULN	26 (37.1)	96 (46.2)	
>ULN	38 (54.3)	75 (36.1)	
Unknown ^1^	6 (8.6)	37 (17.8)	
Symptomatic MBM			0.57
No	19 (27.1)	66 (31.7)	
Yes	51 (72.9)	142 (68.3)	
Previous TTs			*<0.001*
No	49 (70.0)	192 (92.3)	
Yes	21 (30.0)	16 (7.7)	
First line TTs			0.22
No	51 (72.9)	133 (63.9)	
Yes	19 (27.1)	75 (36.1)	
First line ICIs			*<0.001*
No	59 (84.3)	121 (58.2)	
Yes	11 (15.7)	87 (41.8)	
Median time between primary diagnosis of ECM and primary diagnosis of MBM (months) ^2^	8.0 (IQR 6.0–19.0)	7.5 (IQR 3.0–17.0)	0.50

^1^ The “unknown” category was omitted from statistical testing. ^2^ Analyzed for patients with metachronous MBM only (previous TT, *n* = 33; no previous TT, *n* = 71). Abbreviations: ECM: extracranial metastasis, LDH: lactate dehydrogenase, ULN: upper limit of normal (247 U/L), ICIs: immune checkpoint inhibitors, TTs: targeted therapies. Significant *p*-values (<0.05) are in italic.

**Table A6:** Median overall survival of the melanoma-molGPA classes of patients with melanoma brain metastasis (MBM) in the post-2015 cohort with available model input parameters (*n* = 268).

Class	Number of Patients	%	Median OS	HR	*p*-Value
I (0–1)	90	33.6	2.99	ref.	<0.001
II (1,5–2)	116	43.3	6.87	0.627 [0.463–0.849]	
III (2,5–3)	47	17.5	24.51	0.290 [0.189–0.446]	
IV (3,5–4)	15	5.6	30.33	0.203 [0.097–0.423]	
Unknown *	10				

* Patients where the score could not be determined (unknown) were excluded from percentage calculation and analysis.

**Table A7:** A subgroup of 18 patients with melanoma brain metastasis (MBM) who switched from initial targeted therapies (TTs) to immune checkpoint inhibitors (ICIs) after MBM diagnosis.

Variable	At Initiation of TTs *n* = 18 (100%)	At Initiation of ICIs (TTs Switched to ICIs) *n* = 18 (100%)	At First Progression ^2^ after Initiation of ICIs *n* = 18 (100%)
Symptomatic MBM ^1^			
Yes	16 (88.9)	2 (11.1)	7 (38.9)
No	2 (11.1)	13 (72.2)	4 (22.2)
Unknown	0 (0)	3 (16.7)	7 (38.9)
Performance status ^1^			
KPS ≤70	6 (33.3)	2 (11.1)	6 (33.3)
KPS >70	12 (66.7)	15 (83.3)	4 (22.2)
Unknown	0 (0)	1 (5.6)	8 (44.4)
LDH ^1^			
≤ULN	13 (72.2)	10 (55.6)	3 (16.7)
>ULN	3 (16.7)	5 (27.8)	9 (69.2)
Unknown	2 (11.1)	1 (5.6)	6 (33.3)
BM status ^1^			
New/progressive	18 (100)	2 (11.1)	13 (72.2)
Stable/response	0 (0)	12 (66.7)	3 (16.7)
Mixed response	0 (0)	3 (16.7)	1 (5.6)
Unknown	0 (0)	1 (5.6)	1 (5.6)
ECM status ^1^			
New/progressive	15 (83.3)	4 (22.2)	4 (22.2)
Stable/response	1 (5.6)	8 (44.4)	9 (50.0)
Mixed response	0 (0)	2 (11.1)	3 (16.7)
No ECM	2 (11.1)	2 (11.1)	1 (5.6)
Unknown	0 (0)	2 (11.1)	1 (5.6)
Systemic treatment			
Dabrafenib + trametinib	8 (44.4)	NA	7 (38.9)
Vemurafenib + cobimetinib	7 (38.9)	NA	2 (11.1)
Dabrafenib	2 (11.1)	NA	0 (0)
Vemurafenib	1 (5.6)	NA	0 (0)
Encorafenib + binimetinib	0 (0)	NA	3 (16.7)
Nivolumab + ipilimumab	NA	12 (66.7)	NA
Pembrolizumab	NA	4 (22.2)	NA
Nivolumab	NA	2 (11.1)	NA
No systemic treatment	NA	NA	6 (33.3)

^1^ Data were collected when found within +/− two weeks of initiation of TTs, initiation of ICIs, and first progression after start of ICIs, respectively. If not within that time frame, data were regarded as ‘unknown’. ^2^ First progression was defined as first imaging during treatment follow-up with clinician-assessed progression. Abbreviations: LDH: lactate dehydrogenase, ULN: upper limit of normal (247 U/L), BM: brain metastasis, ECM: extracranial metastasis.

### Detailed Description Table A6: Switching from TT to ICI Treatment

Eighteen of four hundred and thirty (4.2%) patients of the total cohort, all with a diagnosis of melanoma brain metastasis (MBM) post-2015, initiated with targeted therapies (TTs) directly after diagnosis of MBM and later switched to immune checkpoint inhibitors (ICIs), after a generally improved clinical condition and/or improving disease status on imaging (Table A6). In 8 of these 18 (44.4%) patients, TTs were combined with surgical resection plus radiotherapy (SRT *n* = 2, WBRT *n* = 1), surgical resection alone (*n* = 2), and radiotherapy alone (SRT *n* = 2, WBRT *n* = 1). Median time between diagnosis of MBM and the start of TTs was 0.76 months. Patients switched to ICIs after a median time of 6.11 months (IQR 3.9–7.0) since the start of TTs. In all 18 patients, TTs were discontinued within one week prior to the start of ICIs.

In three (16.7%) patients, the switch from TTs to ICIs was successful since they had stable disease since ICI initiation until at least data cut-off. These three patients had a median survival time of 25.5 months (IQR 21.7–27.2) since ICI initiation.

Thirteen of eighteen (72.2%) patients had intracranial disease progression after ICI initiation, and four of eighteen (22.2%) patients had extracranial disease progression. Ultimately, 12 of 18 (66.7%) patients switched back from ICIs to TTs at a median time of 2.6 months (IQR 2.5–4.8) since ICI initiation. These 12 patients had a median OS of 11.6 months (IQR 6.5–25.5) since ICI initiation.

Three of eighteen (16.7%) patients progressed after ICI initiation, receiving no further treatment due to poor clinical condition: two patients died of progressive ECM (stable MBM) and one patient of combined progressive MBM and ECM. These three patients had a median OS of 11.9 months (IQR 7.6–16.3) since ICI initiation.
